# Obstructed Hemivagina and Ipsilateral Renal Anomaly (OHVIRA) Syndrome: A Diagnosis in the First Year of Life

**DOI:** 10.7759/cureus.77265

**Published:** 2025-01-11

**Authors:** Carolina Quintela, Ana Sofia Figueiredo, Andreia Dias, António Trindade

**Affiliations:** 1 Pediatrics and Neonatology, Unidade Local de Saúde de Trás-os-Montes e Alto Douro, Vila Real, PRT

**Keywords:** didelphys uterus, obstructed hemivagina, ohvira syndrome, renal anomaly, solitary kidney

## Abstract

Obstructed hemivagina and ipsilateral renal anomaly (OHVIRA) is a rare congenital anomaly that affects females. Although it is possible to diagnose in early life, most cases are usually diagnosed in adolescence or adulthood. This delay in diagnosis can cause a variety of complications, some associated with infertility or renal failure. Here, we report the case of a female child with unilateral renal agenesis identified on the prenatal scans. Prompt postnatal imaging made it possible to diagnose the concomitant gynecological malformation during the first year of life. This report highlights the importance of an early diagnosis in such cases, which could be done through routine screening for gynecological anomalies in patients with prenatal diagnosis of renal malformations.

## Introduction

Obstructed hemivagina and ipsilateral renal anomaly (OHVIRA) syndrome, or Herlyn-Werner-Wünderlich syndrome, is a rare congenital condition of the female genitourinary tract, which results from abnormal development of the Mullerian (paramesonephric) and Wolffian (mesonephric) ducts [1].

Defined by a triad consisting of a didelphys uterus, an obstructed hemivagina, and an ipsilateral renal anomaly, this diagnosis is rarely made before puberty. Most commonly, the diagnosis is made after menarche, which frequently occurs with absent external bleeding and in association with cyclic abdominal pain [1,2]. Nonetheless, in some cases, the diagnosis is achieved during the follow-up of renal anomalies detected in the prenatal period [3]. We present a case diagnosed prepubertally.

## Case presentation

A one-month-old female infant was sent to a pediatric nephrology consultation following a prenatal diagnosis of right renal agenesis. She was the first child of nonconsanguineous parents with an unremarkable family history. The pregnancy was adequately surveilled, with no mention of analytical alterations. Fetal right renal agenesis was first noticed during the third trimester ultrasound performed at 32 weeks and six days of gestation.

Analytically, at birth, the newborn had normal renal function. A renal ultrasound performed in the first 48 hours of life failed to visualize the right kidney, whereas the left kidney was normal. The absence of the right kidney was confirmed by dimercaptosuccinic acid (DMSA) scintigraphy.

**Figure 1:**
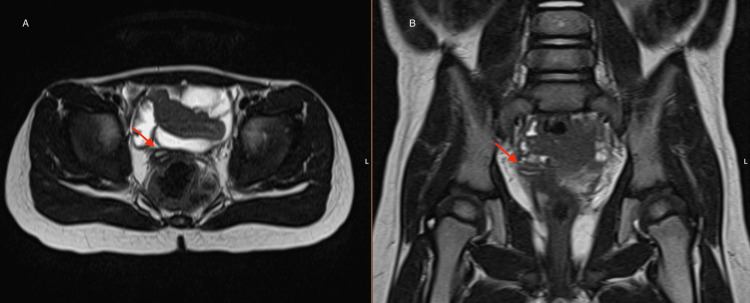
Pelvic MRI confirming OHVIRA syndrome A: Axial plane. B: Coronal plane. MRI: magnetic resonance imaging, OHVIRA: obstructed hemivagina and ipsilateral renal anomaly

At three months of age, the infant underwent a renopelvic ultrasound to screen for associated malformations of the urogenital tract. In the pelvic cavity, two uterine horns with a double cervix were identified, suggesting a uterine didelphys, as well as two hemivaginas, one with liquid distention of its cavity, reaching about 8 mm in caliber, with a suggestive image of a septum. Both ovaries were normal. These findings suggested the diagnosis of OHVIRA syndrome, which was confirmed through magnetic resonance imaging (MRI) (Figure 1).

Presently, the patient is five years old and remains asymptomatic. She maintains regular follow-ups with a pediatric urologist.

## Discussion

The exact incidence of OHVIRA syndrome is still unknown, as there are only a few published case reports [4]. It is supposed that the pathogenesis of this syndrome is explained by the abnormal embryologic arrest of the Mullerian and Wolfian ducts, which occurs around the eighth week of gestation [5]. The Wolfian ducts would normally give origin to the kidneys. Their abnormal development may cause unilateral renal agenesis, as well as an imperforated hemivagina. They also induce the normal development of Mullerian ducts, which are responsible for the development of the uterus and cervix, leading to uterine didelphys and bicollis when their fusion fails [4]. The most common renal anomaly associated with OHVIRA is ipsilateral renal agenesis on the obstructed hemivaginal side; however, other renal anomalies have also been reported, such as pelvic or dysplastic kidneys, and ectopic ureters [2].

The clinical presentation varies widely in symptoms and age of onset [5]. Typically, patients are asymptomatic prior to puberty, when they develop dysmenorrhea and cyclic pelvic pain caused by hematometrocolpos resulting from the obstructed hemivagina [6]. They can also present with a pelvic mass and, less commonly, with abdominal pain, urine retention, vomiting, fever, or vaginal discharge [1]. In the postnatal period and early infancy, due to the influence of maternal hormones, the classic symptoms may also occur [6]. In nonclassical cases, patients may be detected on prenatal ultrasound with a solitary kidney or other renal anomalies. Subsequent postnatal follow-up may reveal additional pelvic abnormalities, leading to further investigation, as in the case presented here [2].

As acute complications, patients with OHVIRA syndrome may present with pyohematocolpos and pyosalpinx. Endometriosis, pelvic inflammatory disease, and infertility are described as possible long-term complications [6]. Also, there is an increased risk of hypertension, proteinuria, and chronic kidney disease [7].

Early diagnosis is important to relieve symptoms and avoid complications. However, it is challenging, particularly during young childhood, given the small size of the gynecological structures, which makes their proper imaging evaluation difficult [1,5]. An ultrasound scan is commonly the first examination performed when investigating pelvic symptoms. However, to evaluate Mullerian duct abnormalities, an MRI is the preferred imaging modality, since it allows a better definition of the type of uterine anomaly and vaginal septum to support a more precise surgical plan [4,5].

Treatment is symptomatic and involves resecting the vaginal septum to enable drainage of the obstructed hemivagina [1]. Hemi-hysterectomy is not indicated [6]. Renal function should be monitored as it can decline around puberty. Patients should be advised to avoid any factors that may affect renal function, such as nonsteroidal anti-inflammatory drugs [7].

## Conclusions

OHVIRA syndrome is a rare condition, with a varied presentation, that requires a high index of suspicion for diagnosis. Early recognition and treatment may prevent long-term urogynecology complications that can cause fertility and renal impairments. Since nowadays, renal anomalies are usually diagnosed prenatally and are routinely confirmed and monitored with postnatal ultrasounds, screening for associated gynecological anomalies is a simple addition that should be routinely performed.
